# Recurrent ectopic pregnancy as a unique clinical sub group: a case control study

**DOI:** 10.1186/s40064-016-1798-0

**Published:** 2016-03-03

**Authors:** Alice Hurrell, Oliver Reeba, Odejinmi Funlayo

**Affiliations:** Department of Gynaecology, Whipps Cross University Hospital, Barts Health Trust, Whipps Cross Road, Leytonstone, London, E11 1NR UK

**Keywords:** Ectopic pregnancy, Diagnosis, Recurrent ectopic pregnancy, Laparoscopy

## Abstract

**Background:**

Women with recurrent ectopic pregnancy (EP) represent a unique cohort of patients in whom diagnostic expertise is paramount. We determined whether recurrent EP is associated with significant differences in patient demographics, clinical presentation, risk factors and surgical findings when compared with primary EP.

**Methods:**

A retrospective case–control study of all EPs diagnosed from 2003 to 2014, at Whipps Cross University Hospital, London.

**Results:**

In the above period 849 EPs were surgically managed (758 primary EPs and 91 recurrent EPs). Recurrent EPs were significantly older than primary EPs (32.2 ± 5.08 vs. 30.5 ± 5.83 years, p < 0.05). They presented at a significantly earlier gestation (5.99 ± 1.08 vs. 6.52 ± 1.81 weeks, p < 0.05) and with a significantly lower primary βHCG (3176 ± 7350 vs. 6243 ± 12,282, p < 0.05). Recurrent EPs were significantly more likely to have a positive history of tubal or pelvic surgery (61.5 % vs. 3.5 %, p < 0.05 and 53.8 vs. 14 %, p < 0.05). At surgery, primary EPs had a significantly greater volume of hemoperitoneum (592 ± 850 vs. 249 ± 391 ml, p < 0.05), whereas recurrent EPs were significantly more likely to have contralateral pathology (31.1 vs. 9.8 %, p < 0.05). Regression analysis showed that the parameters of age, gestational age at presentation, first βHCG level, positive history of previous tubal surgery and previous ectopic pregnancy differ in women at risk of a recurrent EP when compared to women not at risk of a recurrent ectopic (AUC, 0.844).

**Conclusions:**

We conclude that recurrent EPs may represent a unique sub-group of patients with EP.

## Background

An ectopic pregnancy is any pregnancy that implants outside the uterine cavity. The vast majority of ectopic pregnancies implant in the fallopian tube. Despite efforts at primary prevention, the incidence of EP has been stable over recent years, at 11.1 per 1000 pregnancies (The Management of Tubal Pregnancy 2004) though there has been a significant improvement in the mortality from EP as demonstrated in the last UK triennial report (Saving Mothers’ Lives 2006). Creanga et al. estimated trends in EP mortality and examined characteristics of recently hospitalized women who died as a result of EP in the United States and found that the EP mortality ratio declined by 56.6 %, from 1.15 to 0.50 deaths per 100,000 live births between 1980–1984 and 2003–2007 (Creanga et al. 2011).

Despite the declining mortality ratio, being diagnosed with an EP proves an anxiety-provoking time for any woman. In addition to the loss of the pregnancy, there may be acute and chronic implications for both general and reproductive health. All identified risk factors in the many multiple studies are to date maternal: *Chlamydia trachomatis* infection, pelvic inflammatory disease, smoking, tubal surgery, assisted reproductive techniques, previous miscarriages, previous dilatation and curettage and endometriosis. The risk of recurrent EP is greatly increased compared to that of primary EP and reported incidence ranges from 6 to 18 %, with a trend towards higher rates of recurrence after salpingotomy (Jurkovic and Wilkinson 2011; Ankum et al. 1996; Schoen and Nowak 1975). In spite of its identification as being contributory to risk the incidence and occurrence of recurrent EP has not been assessed in research. The challenge of secondary prevention is related to a paucity of modifiable risk factors.

The purpose of our study is to determine whether there is a significant difference in patient demographics, clinical presentation, risk factors and surgical findings between women presenting with primary or recurrent EP. If present, this may help to pre operatively identify women likely to have a recurrent EP.

## Objectives

To determine whether recurrent EP is associated with any significant differences in patient demographics, clinical presentation, risk factors and surgical findings when compared to primary EP.

## Methods

### Study design

A retrospective case–control study of all EPs diagnosed from January 2003 to July 2014, at Whipps Cross University Hospital, London. Data for each surgically managed case of EP presenting to the hospital was entered prospectively into a dedicated database. Exclusion criteria included: non-surgically managed EP and incomplete dataset.

Patient details were collated and variables were collected as below in Table 1.

**Table 1:** Patient details and variables

Variable	Type of variable	Primary [P]/secondary [S]	Description	Units of measurement [P present, A absent]
Age	Demographic characteristic	P	Age of each patient	Years
Parity	Demographic characteristic	P	No of deliveries	Number
Gestation	Demographic characteristic	P	Number of weeks of pregnancy	Weeks
Pain	Clinical characteristic	P	Presenting with abdominal pain	P/A
Vomiting	Clinical characteristic	P	In association with the other symptoms	P/A
Bleeding	Clinical characteristic	P	Vaginal bleeding	P/A
Diarrhoea	Clinical characteristic	P	In association with the other symptoms	P/A
Shoulder tip pain	Clinical characteristic	P	in association with the other symptoms	P/A
Syncope	Clinical characteristic	P	In association with the other symptoms	P/A
Smoking	Risk factor	P	Known long term smoking	P/A
Previous tubal surgery	Risk factor	P	Due to previous tubal pathology	P/A
Previous pelvic surgery	Risk factor	P	Due to previous pelvic pathology	P/A
Previous miscarriage	Risk factor	P	Diagnosed as miscarriage by scan and histopathology	P/A
Previous termination of pregnancy	Risk factor	P	Diagnosed as intra uterine pregnancy by scan and histopathology	P/A
Assisted reproduction	Risk factor	P	Previous pathology	P/A
Previous infertility	Risk factor	P	Previous pathology	P/A
Pelvic inflammatory disease	Risk factor	P	Previous pathology	P/A
Use of intrauterine contraceptive device	Risk factor	P	As contraceptive	P/A
Initial beta HCG level	Investigation	S	Blood test	mIU/ml
Transvaginal/trans-abdominal scan	Investigation	S	Ultrasound scan	Performed/not performed
Adnexal mass	Investigation	S	Scan finding	P/A
Gestational sac	Investigation	S	Scan finding	P/A
Fetal heartbeat	Investigation	S	Scan finding	P/A
Fluid in Pouch of Douglas	Investigation	S	Scan finding	P/A
Empty uterus	Investigation	S	Scan finding	P/A
Hemoperitoneum	Investigation	S	Scan findings	P/A
Laparotomy	Treatment	S	Intra operative findings	P/A
Tubal ectopic pregnancy	Treatment	S	Intra operative findings	P/A
Salpingectomy	Treatment	S	Intra operative findings	P/A
Salpingotomy	Treatment	S	Intra operative findings	P/A
Hemoperitoneum	Treatment	S	Intra operative findings	P/A
Presence of hepatic adhesions	Treatment	S	Intra operative findings	P/A
Contralateral pathology	Treatment	S	Intra operative findings	P/A

### Statistical analysis

Data was collected and stored on an Excel spreadsheet. Caldicott guidelines were strictly adhering to in the collection and storage of personal information (Crook 2003). Results were analyzed and compared using the Student’s t test and Fisher’s test. Parametric tests were used, as all data passed the test of normality. Student’s t test was used for numerical data and Fisher’s exact test for categorical data. Significance was set at a p value of <0.05. Multivariate logistic regression analysis was performed using the significant variables from the univariate analysis. Analysis was performed using Graphpad Prism, Version 6.0 (Graphpad software, San Diego, USA).

## Results

There were 849 consecutive EPs diagnosed and managed surgically from January 2003 to July 2014. Of these, 758 were primary EPs and 91 were recurrent EPs. Of these 52, 6.2 % of the study cohort was not tubal EPs: 29 cornual and one repeat cornual EP, 19 ovarian with no recurrent EPs. There were 1 peritoneal, 2 CS scar ectopics, 1 heterotropic and 1 rudimentary horn pregnancy. Non tubal and tubal EPs were analysed together.

Women with recurrent EPs were significantly older than women with primary EPs (32.2 ± 5.08 vs. 30.5 ± 5.83 years, p < 0.05). They presented at a significantly earlier gestation (5.99 ± 1.08 vs. 6.52 ± 1.81 weeks gestation, p < 0.05) and with a significantly lower primary βHCG (3176 ± 7350 vs. 6243 ± 12,282 IU/l, p < 0.05) (Table 2).

**Table 2:** The comparison of the baseline characteristics

	Recurrent ectopic	Primary ectopic			
	Mean (±SD) or % (n)	Mean (±SD) or % (n)	p value	Mean difference or odds ratio	95 % CI
Baseline characteristics of women with EP					
Age^a^	32.2 (5.08)	30.5 (5.83)	0.0049	−1.65	−2.78 to −0.51
Parity 1^b^					
P0	37.5 (33/88)	44.7 (327/732)	0.2127	0.7431	0.471 to 1.172
P1–P3	54.5 (48/88)	51.8 (379/732)	0.6526	1.118	0.717 to 1.742
P4 or more	8.0 (7/88)	3.6 (26/732)	0.0755	2.347	0.9872 to 5.578
Gestation^a^	5.99 (1.08)	6.52 (1.81)	0.0001	0.5402	0.2728 to 0.8076
Risk factors for EP in women with primary and recurrent EP					
Smoking	18.7 (17/91)	14.6 (108/741)	0.3498	1.346	0.7650 to 2.370
Previous tubal surgery	61.4 (54/88)	3.5 (26/740)	<0.0001	43.62	24.40 to 77.97
Previous pelvic surgery	53.8 (49/91)	14.0 (104/743)	<0.0001	7.168	4.519 to 11.37
Previous miscarriage	27.5 (25/91)	24.1 (179/742)	0.5185	1.191	0.7297 to 1.945
Previous TOP	14.3 (13/91)	18.7 (139/743)	0.3875	0.7242	0.3913 to 1.340
Assisted reproduction	3.3 (3/91)	4.3 (32/743)	1.0	0.7575	0.2272 to 2.526
Previous infertility	12.1 (11/91)	7.8 (58/743)	0.1602	1.624	0.8186 to 3.222
Previous PID	8.8 (8/91)	7.7 (57/742)	0.6794	1.158	0.5339 to 2.513
IUD	0 %	1.3 % (10/733)	0.6121	2.62	0.1521 to 45.11
Mirena coil	0 %	0.9 % (7/743)	1	1.864	0.1055 to 32.92
Clinical presentation of women with primary and recurrent EP					
Pain^b^	93.4 (85/91)	94.5 (705/746)	0.63	0.8239	0.3397 to 1.998
Bleeding^b^	80.2 (73/91)	85.0 (634/746)	0.2239	0.7164	0.4118 to 1.246
Vomiting^b^	7.7 (7/91)	9.7 (72/746)	0.7041	0.7801	0.3475 to 1.751
Diarrhoea^b^	0 (0/91)	1.9 (14/746)	0.3849	0.2761	0.01632 to 4.670
Shoulder tip pain^b^	4.4 (4/91)	10.5 (78/745)	0.0899	0.3932	0.1404 to 1.101
Syncopal attack^b^	6.6 (6/91)	11.1 (83/745)	0.2112	0.563	0.2385 to 1.329
1st βHCG^a^	3176 (7350)	6243 (12,282)	0.0099	3053	750 to 5355
TVS^b^	95.6 (87/91)	88.3 (651/737)	0.0323	0.348	0.1246 to 0.9724
TAS^b^	12.1 (11/91)	17.1 (126/738)	0.2943	1.497	0.7747 to 2.894
Both TVS and TAS^b^	11.0 (10/91)	12.0 (88/735)	1	1.102	0.5505 to 2.205
Ultrasound scan findings					
Adnexal mass^b^	84.6 (77/91)	88.6 (643/726)	0.3007	1.409	0.7624 to 2.602
Gest sac^b^	24.2 (22/91)	20.3 (148/730)	0.4104	0.7976	0.4776 to 1.332
FH^b^	12.1 (11/91)	8.9 (65/730)	0.3363	0.7109	0.3602 to 1.403
PoD fluid^b^	68.1 (62/91)	76.3 (557/730)	0.094	1.506	0.9385 to 2.417

The comparison of the baseline characteristics, risk factors, the clinical presentations and ultrasound scan findings of women presenting with primary EP compared with recurrent EP

*TVS* transvaginal ultrasound scan, *TAS* transabdominal ultrasound scan, *PID* pelvic inflammatory disease, *IUD* intrauterine device, *FH* fetal heartbeat, *PoD* pouch of douglas

^a^Data shown as mean (SD) and analysed by Student’s t test, with mean difference and 95 % confidence interval

^b^Data shown as % and analysed by Fisher’s exact test with Odds ratio (OR) and 95 % confidence interval

There was no significant difference in symptoms at the time of clinical presentation between women with primary or recurrent EP. However, there was a non-significant trend towards a greater proportion of patients with primary EP presenting with shoulder tip pain (10.5 % of primary EPs vs. 4.4 % of recurrent EPs, p = 0.089) (Table 2). This is presumably in keeping with women with primary EPs presenting with a significantly greater volume of hemoperitoneum (see Table 3). Women with recurrent EPs had a significantly lower primary βHCG, in association with the significantly earlier gestation at time of clinical presentation (Table 2). Women with recurrent EP were also significantly more likely to have a transvaginal ultrasound scan.

**Table 3:** Operative findings

	Recurrent ectopic	Primary ectopic			
	% (n)	% (n)	p value	Mean difference or odds ratio	95 % CI
Presence of haemoperitoneum^a^	89.9 (80/89)	89.8 (654/728)	1	1.006	0.4847–2.087
Volume of haemoperitoneum (mls)^b^	249 (391)	592 (850)	<0.0001	360.6	249.7–471.4
Presence of hepatic adhesions^a^	13.5 (12/89)	7.6 (53/695)	0.0666	1.888	0.9661–3.689
Contralateral pathology^a^	31.1 (28/90)	9.8 (74/755)	<0.0001	4.156	2.504–6.899
Laparotomy^a^	1.1 (1/90)	4.5 (34/756)	0.1642	0.2386	0.03225–1.765
Tubal ectopic^a^	97.8 (89/91)	93.3 (705/756)	0.1082	3.219	0.7702–13.46
Salpingectomy^a^	57.1 (52/91)	83.3 (625/750)	<0.0001	0.2667	0.1688–0.4214
Salpingotomy^a^	37.4 (34/91)	10.9 (82/750)	<0.0001	4.859	2.998–7.875

The intra operative findings of women presenting with primary EP compared with recurrent EP

^a^Data shown as  % and analysed by Fisher’s exact test with odds ratio (OR) and 95 % confidence interval

^b^Data shown as mean (SD) and analysed by Student’s t test, with mean difference and 95 % confidence interval

Women with recurrent EP were unsurprisingly significantly more likely to have had previous tubal surgery or previous pelvic surgery (Table 2). However, there was otherwise no significant difference in risk factors between women presenting with recurrent or primary EP. It is noteworthy that women with recurrent EP were not significantly more likely to have had previous pelvic inflammatory disease (Table 2).

There were no significant differences in scan findings between the two sub-groups (Table 2). At the time of surgery, primary EP was significantly associated with greater volume of hemoperitoneum (592 ± 850 vs. 249 ± 391 ml, p < 0.05). Women with recurrent EP were significantly more likely to have contralateral pathology (31.1 vs. 9.8 %, p < 0.05) and there was a non-significant trend towards a greater proportion of peri-hepatic adhesions (13.5 vs. 7.6 %, p = 0.066). In association with the desire to preserve future fertility, women with recurrent EP were significantly less likely to have a salpingectomy (57.1 vs. 83.3 %, p < 0.05) and significantly more likely to have a salpingotomy (37.4 vs. 10.9 %, p < 0.05) (see Table 3).

**Fig. 1:**
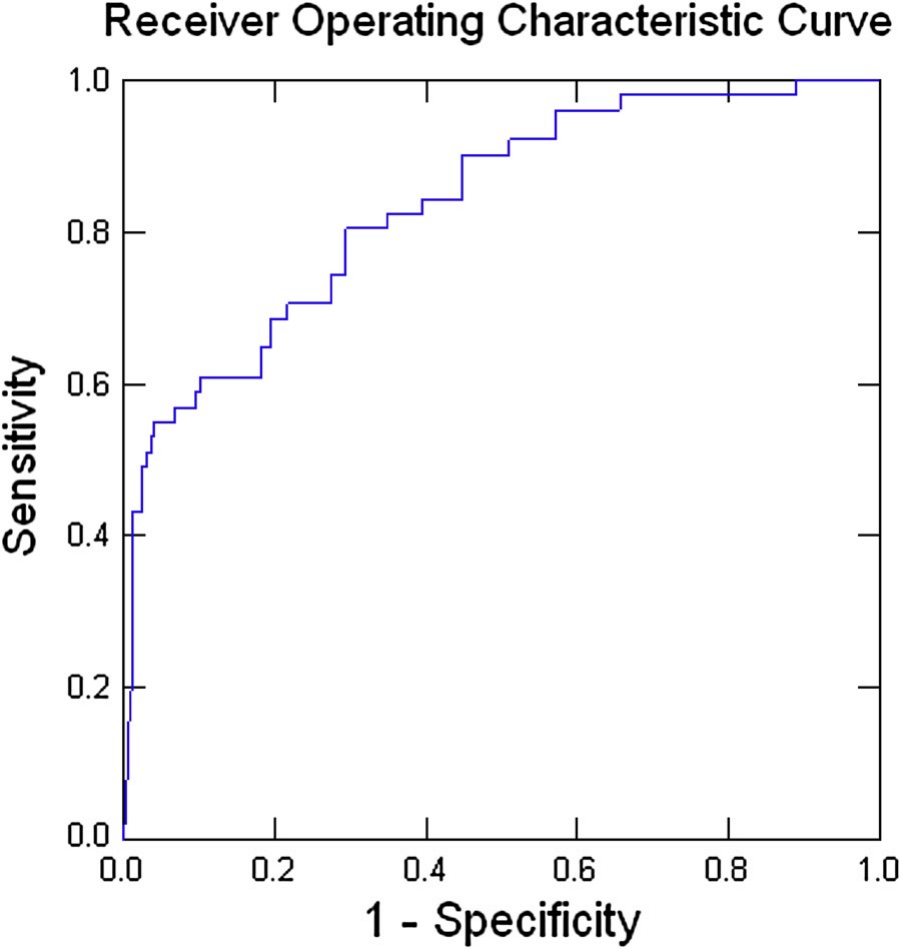
Area under the curve AUC, 0.844. It shows the ROC curve obtained from the predicted probabilities of the logistic regression

The significant preoperative factors from the univariate analysis were entered into a logistic regression analysis. The predicted probabilities from the same were used to construct a ROC curve (Fig. 1). The AUC calculated was 0.844, which indicates good differentiation between the two groups. Thus the parameters of age, gestational age at presentation, level of first βHCG, positive history of previous tubal surgery and previous EP differ in women at risk of a recurrent EP when compared to women not at risk of a recurrent ectopic (Table 4).

**Table 4:** Logistic regression analysis

Parameter	Odds ratio	STD error	p value	95 % CI	
				Lower	Upper
Age	1.085	0.033	0.008	0.021	0.142
Gestation	0.808	0.101	0.090	−0.459	0.033
Previous tubal surgery	21.133	9.473	0.000	2.172	3.929
Previous pelvic surgery	2.455	1.008	0.029	0.093	1.703
First βHCG	1.000	0.000	0.058	0.000	0.000

The logistic regression analysis results of the variables which were significant of univariate analysis

## Discussion

Our study shows that women with recurrent EP may represent a distinct subgroup of patients with EP. This is particularly in relation to awareness of the condition and early presentation, as they are significantly more likely to present at an earlier gestation, with a lower βHCG and significantly less hemoperitoneum. Universally, women treated for EP are informed of the approximately 10 % risk of recurrence of EP and they are advised to have an early pregnancy ultrasound scan in any future pregnancy. Our results contrast with a previous study of recurrent EP, which found a non-significant trend towards a higher βHCG (Butts et al. 2003). These contrasting results may represent our thorough post-operative counseling and follow up. The lower βHCG level may reflect an earlier presentation of the women due to our stringent follow up protocol and counseling. Women are seen post operatively in a dedicated clinic or are telephoned in our dedicated telephone clinic and debriefed about their diagnosis and treatment. They are advised on the clinical course to follow with the next pregnancy, including getting in touch with their general practitioner as soon as the pregnancy test becomes positive. An early ultrasound scan is arranged to facilitate early diagnosis. This indicates that women are being diagnosed at an earlier stage when more tubal preservation could take place.

Women with recurrent EP were significantly older and a greater proportion was parous, although this did not reach statistically significance. This is consistent with the study by Butts et al. (Butts et al. 2003). In the UK, parous women are excluded from assisted conception treatment on the National Health Service and therefore everything should be done to preserve the affected tube, with the necessary surgical expertise available for management (Fertility: assessment and treatment for people with fertility problems 2013; Odejinmi et al. 2008). The ESEP study found that in women with a tubal pregnancy and a healthy contralateral tube, salpingotomy does not significantly improve fertility prospects compared with salpingectomy (Mol et al. 2014). Though the limitations of the ESEP study should be taken into consideration when managing these groups of women (Odejinmi 2014).

Unsurprisingly, women with EP were significantly more likely to have contralateral tubal pathology. There has always been some debate as to what type of surgery should be performed in women with a damaged contralateral tube (The Management of Tubal Pregnancy 2004). The consensus is that women should be treated on an individual basis with informed consent. If the contralateral tube is damaged and preservation of fertility is a priority, then a salpingotomy is advised, as although this increases the chance of recurrent EP to 20 %, the chance of intrauterine pregnancy is 50 %. Extrapolation from this would mean that if women have a recurrent EP, ideally they should have a salpingotomy, which again needs additional surgical expertise. Although in the short-term a salpingotomy is more expensive, due to additional post-operative follow up and treatment for persistent trophoblast that may become necessary, the reduced need for assisted conception renders this treatment more cost-effective in the long-term (The Management of Tubal Pregnancy 2004; Mol et al. 1997).

The results from our logistic regression analysis of significant pre-operative characteristics emphasize that the groups are significantly different. Women with recurrent EP are more likely to be older, with a positive history of previous tubal or pelvic surgery and to present at an earlier gestation with a lower βHCG. Therefore, it is paramount that transvaginal ultrasonography is performed by a sufficiently experienced practitioner and if surgical management is considered, then the necessary surgical expertise is available (Odejinmi et al. 2008; Brown and Doubilet 1994; Crochet et al. 2013).

It is somewhat surprising that there was no significant difference in the history of previous pelvic inflammatory disease (PID) between the two groups. One might expect that women with recurrent EP would be more likely to have a history of previous PID or any other pathology that would be directly responsible for recurrence. Our finding is consistent with some of the literature (Butts et al. 2003; Joesoef et al. 1991), but contrasts with another large study of recurrent EP, which found that a history of infectious pathology tripled the risk of recurrent EP (Skjeldestad et al. 1998). Possible explanations for this include the fact that sexually transmitted infections and PID are notoriously both under-reported and under-diagnosed (Bouyer et al. 2003). This is one of the facets of contributing risk which was not explored in our study. Our protocol for patient management during investigation and management for an EP included investigations for Chlamydia only if there was a positive history of previous/recent PID. Thus along with the majority of similar studies, actual numbers of infections could have been under reported in our study. In a previous study of EP, although less than 10 % of patients gave a history of PID/salpingitis, over 75 % were found to have antibodies to *N gonorrhoea* or *C trachomatis*, suggesting that infection is commonly sub-clinical (Spandorfer and Barnhart 2003). The link with sub-clinical PID and EP has been evidenced (Sweet and Gibbs 2012). And therefore, it is possible that a significant proportion of our patients had sub-clinical PID and that our results on history of previous PID are therefore unreliable. The significant impact on future reproductive health, including recurrent EP, emphasizes the importance of establishing a history of PID and regularly testing for sexually transmitted infections, even in apparently low risk women.

When considering other established risk factors for EP, there was no statistically significant difference in risk factors between the two groups, except for previous tubal or pelvic surgery. However, there were a marginally higher proportion of smokers, women with a previous miscarriage and women with previous subfertility in the group with recurrent EP. Conversely, there were 17 women out of 758 with a primary EP who had a Mirena or copper coil in situ, compared to 0 % of women with recurrent EPs (again, this did not reach statistical significance). This may represent the fact that women who have already had one EP do not wish to expose themselves to anything else which may increase their risk of ectopic implantation (Bouyer et al. 2003; Bouyer et al. 2000; Mol et al. 1995; Parashi et al. 2014). Women who want to avoid a recurrent EP and need contraception sometimes opt for an implant but this also has its drawbacks as it has been shown that although the failure rate is extremely low the risk of EP is still present (Olowu et al. 2011). The review by Rana et al. showed that surgically managed EPs are decreasing and not the actual incidence of EP (Rana et al. 2013).

Our study is limited by the fact that we have only included women who have had a surgically managed EP. Therefore, we are missing women with a recurrent EP who had conservative or medical management of their EP. At our unit, we have been collecting data on conservative or medical management since 2009—during this period there have been 13 recurrent EPs that were managed with conservative or medical management. Nevertheless, our cohort represents the largest study of recurrent EP in the literature. Expecting a recurrent EP pre operatively is of importance in planning the surgical outcome as many tubal preservation techniques are being used successfully (Watrowski 2014).

The non-tubal as well as the tubal EPs were considered together. The risk factors and etiology of non tubal and tubal implantation are not significantly different. The symptoms and signs of non tubal ectopic pregnancy do not significantly differ from those of tubal pregnancies though they tend to present later. The symptoms are similar and the only significant differences are due to the delayed diagnosis leading to accentuation of the symptoms at presentation (Alalade et al. 2015). Due to all the above factors it was felt that excluding non tubal EPs from the analysis would introduce bias.

One of the limitations of our study is that one of primary ectopic pregnancies if followed up for a further number of years could develop a recurrent ectopic. This bias in our study is limited by the fact that the numbers of primary ectopics are significantly greater than that of the recurrent ectopics. Additionally none of the primary ectopic pregnancies have developed another ectopic since July 2014. A better study design would be a cohort study, including patients who have their first ectopic pregnancy during a specific period of time and subsequently observed during a certain number of years in order to differentiate those patients who have recurrent ectopic pregnancies from those who do not have more ectopic pregnancies.

In conclusion, we have demonstrated that women with recurrent EP represent a unique sub-group of women with EP. They are older, more likely to present at an earlier gestation, with a lower initial βHCG and significantly less hemoperitoneum. We found no significant difference in symptoms at presentation, or in risk factors present—except for a history of tubal or pelvic surgery. Unfortunately, there is a paucity of modifiable risk factors for secondary prevention of recurrent EP. The implications for future reproductive health require women with recurrent EP to be rapidly identified and managed with the appropriate level of surgical expertise.
